# Supplementary material for: Integrated multi-omic analysis reveals novel subtype-specific regulatory interactions in pediatric B-cell acute lymphoblastic leukemia
Source: bioRxiv. 2025 Aug 17:2025.08.13.670107. Preprint. [Version 1] doi: 10.1101/2025.08.13.670107 (PMC12363954; doi:10.1101/2025.08.13.670107)
Supplement: 1 [file NIHPP2025.08.13.670107V1-supplement-1.pdf]

bioRxiv preprint doi: https://doi.org/10.1101/2025.08.13.670107; this version posted August 17, 2025. The copyright holder for this preprint (which was not certified by peer review) is the author/funder, who has granted bioRxiv a license to display the preprint in perpetuity. It is made available under aCC-BY 4.0 International license.

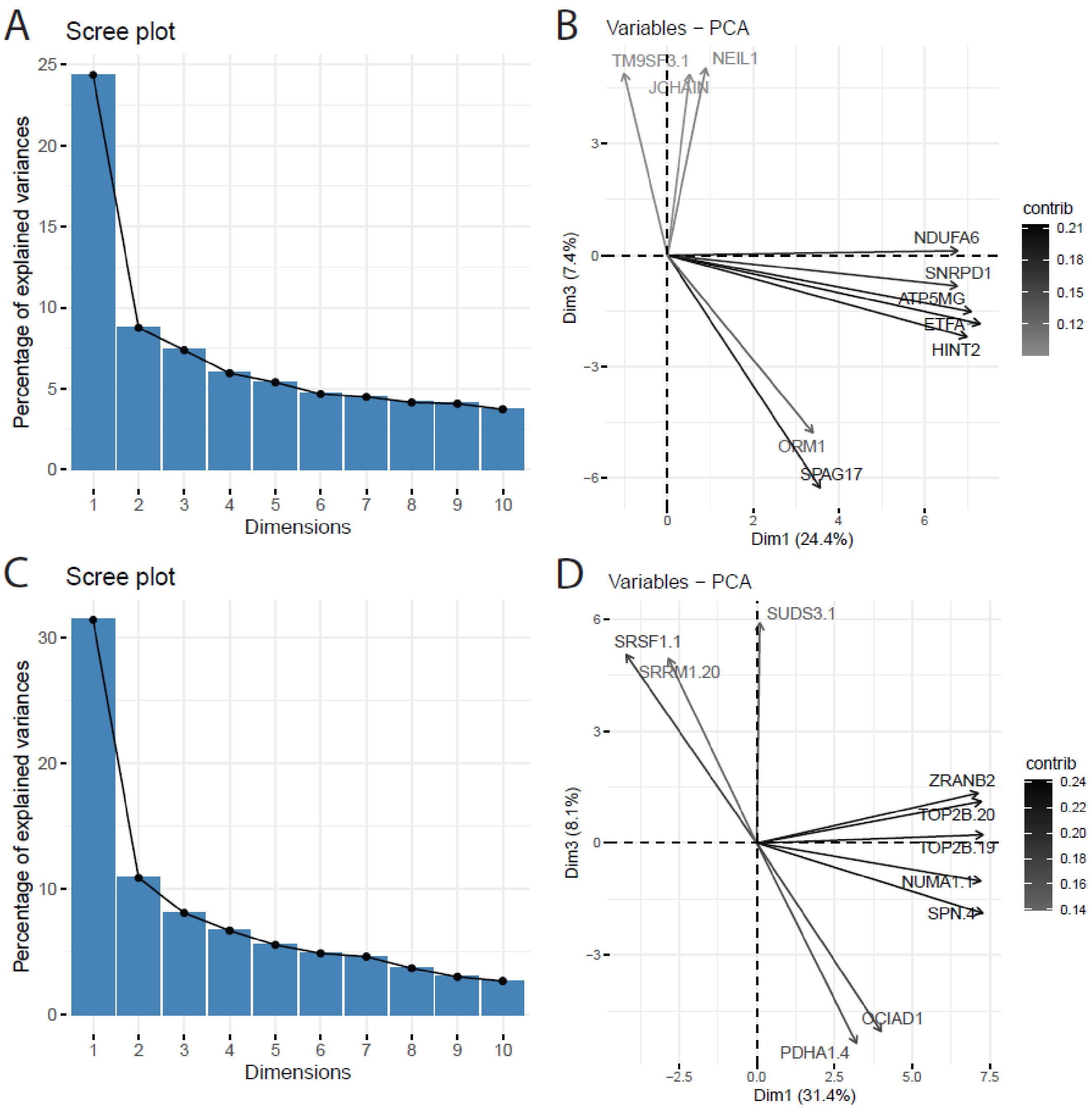

Supplementary Figure 1. Scree plots and top 5 variables driving dimensions 1 and 3 for proteomic (A-B) and phosphoproteomic (C-D) PCAs.
